# Sugar Diet in Intolerant Ulcer of the Stomach

**Published:** 1914-05

**Authors:** 


Sugar Diet in Intolerant Ulcer of the Stomach. Maurice Loeper, Paris, *Le Prog. Med.,* October 4, 1913. The author employs a diet low in proteid and with the addition of about 100 grams of sugar, either as such or in jellies, conserves, etc. This diet is used for about 5 days, glycosuris, if appreciable at all doing no harm. This amount of sugar adds about 400 calories and facilitates both general recuperation and healing of the ulcer. Various tables of nutritive ingredients are given but these are not reproduced as similar tables have been issued by our Government and can be found in several works on dietetics, in more elaborate form. (Note: Having determined by estimates of average diet of persons in ordinary health that the ordinary American dietary contains about 100 grams of sugar a day, in addition to sugar taken between meals in the form of candy, fruits, soft drinks, etc., and having made an estimate of personal dietary as ordinarily containing 150-225 grams of sugar a day, in various forms, we are not much impressed with the designation of this diet as "saccharated." However, the use of sugar in gastric ulcer, after the period of emergence, should be fairly liberal, though Loeper's diet is, from the American standpoint, rather a moderate restriction of the ordinary diet in this respect, than one to which sugar has been intentionally added.)

